# Real-world evidenceand clinical observations of the treatment of advanced non-small cell lung cancer with PD-1/PD-L1 inhibitors

**DOI:** 10.1038/s41598-019-40748-7

**Published:** 2019-03-12

**Authors:** Peng Song, Jingcheng Zhang, Congcong Shang, Li Zhang

**Affiliations:** 1 Department of Respiratory Medicine, Peking Union Medical College Hospital, Chinese Academy of Medical Sciences & Peking Union Medical College, Beijing, China; 2 Department of Internal Medicine, Peking Union Medical College Hospital, Chinese Academy of Medical Sciences & Peking Union Medical College, Beijing, China; 3 Department of allergy, Henan Provincial People’s Hospital, Zhengzhou, China

## Abstract

To summarize the therapeutic effects of PD-1/PD-L1 inhibitors on patients with advanced non-small cell lung cancer (NSCLC) in a real-world setting, we attempted to identify potential molecular biomarkers or clinical factors that reflected the therapeutic effect. The medical records of patients with non-small cell lung cancer who were treated with PD-1/PD-L1 inhibitors were obtained from the outpatient department or inpatient department of Peking Union Medical College Hospital from August 1, 2015, to January 1, 2018. Our follow-up continued until May 1,2018. We chose overall survival (OS) as the primary observation endpoint and progression-free survival (PFS), objective response rate (ORR), disease control rate (DCR), and safety as the secondary observation endpoints. Efficacy was evaluated according to the Response Evaluation Criteria in Solid Tumors (RECIST) 1.1. The Kaplan-Meier method was used to generate survival curves, and we compared the influence of different factors on PFS and OS by the log-rank test. The median follow-up time was 11 months. At the end of the follow-up, 24 patients (61.5%) were still undergoing immunotherapy, and 7 patients (17.9%) had died. Twenty-six cases (66.7%) employed PD-1/PD-L1 inhibitors as first-line treatment, and 7 cases (17.9%) employed PD-1/PD-L1 inhibitors as second-line treatment. Only 6 cases (15.4%) employed PD-1/PD-L1 inhibitors as third-line treatment. Therapeutic effect evaluation: Complete response (CR): 1 case (2.6%). Partial response (PR): 10 cases (25.6%). Stable disease (SD): 16 cases (41.0%). Progressive disease (PD): 12 cases (30.8%). The ORR was 28.2%, and DCR was 69.2%. The median PFS was 25.5 months (95% CI 6.8–44.1 months), which failed to reach the median OS. PD-1/PD-L1 inhibitor treatment is more effective for advanced non-small cell lung cancer patients in a real-world setting than in clinical trials; PD-1/PD-L1 inhibitor treatment is more effective for people who are over 70 than for people who are under 70. Additionally, patients who are over 75 years old have a higher response rate, suggesting that elderly patients may receive more benefits from immunotherapy; Patients who have an epidermal growth factor receptor (EGFR) mutation (+) may benefit from immunotherapy after treatment with a tyrosine kinase inhibitor (TKI). It is essential to identify these potential patients from the entire patient pool; PD-1 may have a certain curative effect on brain metastases from NSCLC. Local radiotherapy may help to improve PD-1 intracranial efficacy.

## Introduction

Lung cancer is a deadly malignant disease that has the highest morbidity and mortality in China. Non-small cell lung cancer (NSCLC) is the most common type of lung cancer (80–85%), and approximately 57% of patients who have NSCLC already had distant metastases when their diagnosis was confirmed^1^. In 2015, there were 733,300 new cases of lung cancer in China and 610,200 lung cancer-related deaths in total^2^. The morbidity of lung cancer has continued to rise in recent years. For patients with advanced non-small cell lung cancer, systemic therapy based on histological subtype was the main treatment method, and first-line treatment was platinum-containing dual-drug chemotherapy. With the identification of lung cancer driver genes such as EGFR, ALK, ROS1, BRAF, molecular targeted therapy has further prolonged progression-free survival and overall survival of patients and has become a first-line treatment option for populations with therapy-sensitive mutations^3^.

As a highly invasive tumour, unfortunately, the five-year survival rate of lung cancer is only approximately 10–20% world-wide. Molecular targeted therapy, though effective, inevitably develops drug resistance over time; thus, it cannot provide long-term survival for all patients. For patients with advanced non-small cell lung cancer without therapy-sensitive mutations, first-line treatment is still based on chemotherapy, which eventually leads to progression of disease. With such a large population with advanced lung cancer, a more effective treatment has become a critical clinical problem that needs to be solved. In recent years, the success of tumour immunotherapy by immune checkpoint inhibitors (ICIs) in the treatment of solid tumours has provided new hope for solving this problem. PD-1/PD-L1 is the most widely used immune checkpoint inhibitor, and other immune checkpoints, such as CTLA-4, TIM-3, LAG-3, and TIGIT, are also being developed. Based on the results of several key phase III clinical trials, the latest National Comprehensive Cancer Network (NCCN) guidelines stated that nivolumab and atezolizumab can be used in patients with non-sensitive mutant NSCLC as second-line treatment and that pembrolizumab can be used as second-line treatment in patients who have PD-L1 expression in ≥1% tumour cells or as first-line treatment in patients who have PD-L1 expression in ≥50% tumour cells^4–8^.

However, it is well known that randomized controlled trials (RCTs) have strict entry requirements to guarantee internal stability, which can lead to the loss of external scalability. People with poor prognosis, such as patients who are older, have an ECOG score of 2 or more, have comorbidities, and have brain metastases, are rarely included in randomized controlled clinical trials. These patients tend to benefit less and are more prone to having toxic reactions to systemic treatment. Therefore, the results of clinical trials do not fully reflect real clinical situations^4–8^. Furthermore, real-world data can make up for the shortcomings of RCTs and further guide follow-up studies after RCTs. As nivolumab and pembrolizumab became available in China in July 2018, Chinese lung cancer specialists need to address the immunotherapy needs of patients with lung cancer in various situations, and they need real-world data on immunotherapy in lung cancer. This study collected data from all of the patients who were treated with immune checkpoint inhibitors at Peking Union Medical College Hospital during our follow-up to summarize the effectiveness of immune checkpoint inhibitors in real-world lung cancer patients and to analyse potential clinical factors associated with prognosis.

## Research Objectives

To summarize the therapeutic effect of PD-1/PD-L1 inhibitors in patients with advanced non-small cell lung cancer in a real-world setting, we attempted to identify potential molecular biomarkers or clinical factors that reflected the therapeutic effect.

## Data and Methods

We obtained the medical records of the patients who received PD-1/PD-L1 inhibitor treatment and underwent follow-up at Peking Union Medical College Hospital from August 2015 to February 2018. The study was approved by the ethics committee of Peking Union Medical College Hospital and complied with the Declaration of Helsinki and good clinical practice guidelines.

### General data

Clinical indicators included sex, date of diagnosis, age at the start of immunotherapy, TNM staging at diagnosis and immunotherapy initiation, pathological type of lung cancer, gene mutation status, molecular pathology of tumour, specimen source for genetic testing or PD-L1 detection, gene mutation and PD-L1 detection method, ECOG score, history of smoking at the start of diagnosis and immunotherapy, brain metastasis status at the time of diagnosis, time to starting treatment, treatment plan, time of progression, start date of immunotherapy, how many treatment lines had been completed prior to the initiation of immunotherapy, immunotherapy cycle, immunotherapy regimen, immune-related side effects, optimal efficacy, time of immunotherapy, and post-immune therapy.

Laboratory tests included routine blood tests, liver and kidney function, tumour markers, T lymphocyte subsets, and thyroid function.

Imaging examination included CT, bone scan, cranial MRI, and PET-CT.

### Treatment plan

Thirty-nine patients received immunotherapy, and 26 patients received it as first-line treatment. Seven patients received immunotherapy as second-line treatment, and 6 patients received it as third-line and above. In regard to therapy used, there are 31 cases that used pembrolizumab, 4 cases that used nivolumab, and 4 cases that used atezolizumab. Routine blood tests, liver and kidney function, and blood Mg were evaluated before each cycle of treatment. Chest imaging was performed every 3 cycles, and treatment was continued until intolerable toxicity or disease progression occurred. Patients who had been shownto benefit from immunotherapy could continue to be treated with PD-1/PD-L1 inhibitors even if disease progression was confirmed radiographically.

### Evaluation of efficacy

According to the Response Evaluation Criteria in Solid Tumors (RECIST 1.1)^9^, the efficacy evaluation standard is divided into complete response (CR), partial response (PR), stable disease (SD) and progressive disease (PD). Pseudo-progression was defined as a decrease of the total size of tumour target lesions by more than 30% from baseline after PD was assessed by RECIST 1.1. Objective response rate (ORR) was defined as the percentage of patients with CR + PR among the patients, and the disease control rate (DCR) was defined as the percentage patients with CR + PR + SD among all patients. Progression-free survival (PFS) referred to the time from receiving the first dose of PD-1/PD-L1 inhibitor treatment to PD or death. Overall survival (OS) referred to the time from receiving the first dose of PD-1/PD-L1 inhibitor treatment to death or the end of the observation.

### Statistical analysis

Categorical variables were analysed as percentages, and continuous variables were analysed as medians. Statistical analysis was performed by SPSS 19.0 statistical software, and ORR and DCR were calculated. Fisher’s exact test was used to analyse short-term efficacy and clinical features. PFS and OS were analysed by the Kaplan-Meier method and log-rank test. P < 0.05 was considered to be statistically significant. To account for the small sample size and categorical variables, this study used the random forest (RF) method to utilize random resampling techniques (bootstrap aggregating) and the random subspace method to construct multiple decision trees and obtain the final classification result by voting. Random forests can be used to rank the importance of variables in a regression or classification problem in a natural way, so this step can greatly reduce the limitations of traditional Fisher’s test methods in classification studies.

### Informed consent

Informed consent was obtained from all individual participants included in the study.

## Results

### Baseline characteristics

A total of 43 patients with non-small cell lung cancer received PD-1/PD-L1 treatment. One patient who was not evaluated for efficacy and three patients who were lost to follow-up were excluded. Thus, thirty-nine patients were included in the study. The median age of the population was 62 years (32–83 years). Ten patients (25.6%) were over 70-years-old, and 6 patients were over 75-years-old (15.3%). There were 28 males (71.8%) and 11 females (28.2%). The ratio of adenocarcinoma (46.1%) to squamous cell carcinoma (48.7%) was approximately equal. We found that most patients had a history of smoking (69.2%), and most patients had stage IV disease (74.3%) during immunotherapy. The number of patients who received immunotherapy as first-line treatment was 26 (66.7%) in our study. In regard to metastases, 5 patients (12.8%) had intracranial metastases before immunotherapy. There were four patients whose EGFR gene mutation status was evaluated, there were three patients (75%) with EGFR therapy-sensitive mutations and there was one patient (25%) with a non-sensitive mutation. In addition, three patients had previously received autologous lymphocyte reinfusion therapy before immunotherapy. The baseline characteristics are shown in Table 1.

**Table 1:** Baseline characteristics (n = 39).

Age (median, range)	62 (34–83) N (%)
**Sex**	
Male	28 (71.8%)
Female	11 (28.2%)
**Histological subtype**	
Adenocarcinoma	18 (46.1%)
Squamous cell carcinoma	19 (48.7%)
Adenosquamous carcinoma	2(5.1%)
**ECOG PS score at the time of pembrolizumab initiation**	
0	17 (43.6%)
1	19 (48.7%)
2	3 (7.7%)
**Stage at the time of pembrolizumab initiation**	
IIIB	3(7.7%)
IIIC	8(20.5%)
IVA	18 (46.2%)
IVB	10(25.6%)
**Smoking history**	
never	12 (30.7%)
yes	27 (69.2%)
**Pembrolizumab initiation (line of therapy)**	
1	26 (66.7%)
2	7 (17.9%)
≥3	6 (15.4%)
**PD-L1 status**	
positive	16 (41.0%)
Negative/Unknown	23 (59.0%)
**EGFR Genetic testing (non-squamous cell carcinoma**, **n = 4)**	
EGFR exon 19 deletion	1 (25%)
EGFR exon21 L858R	2 (50%)
EGFR exon 21 L861Q	1 (25%)

### Follow-up

**Figure 1:**
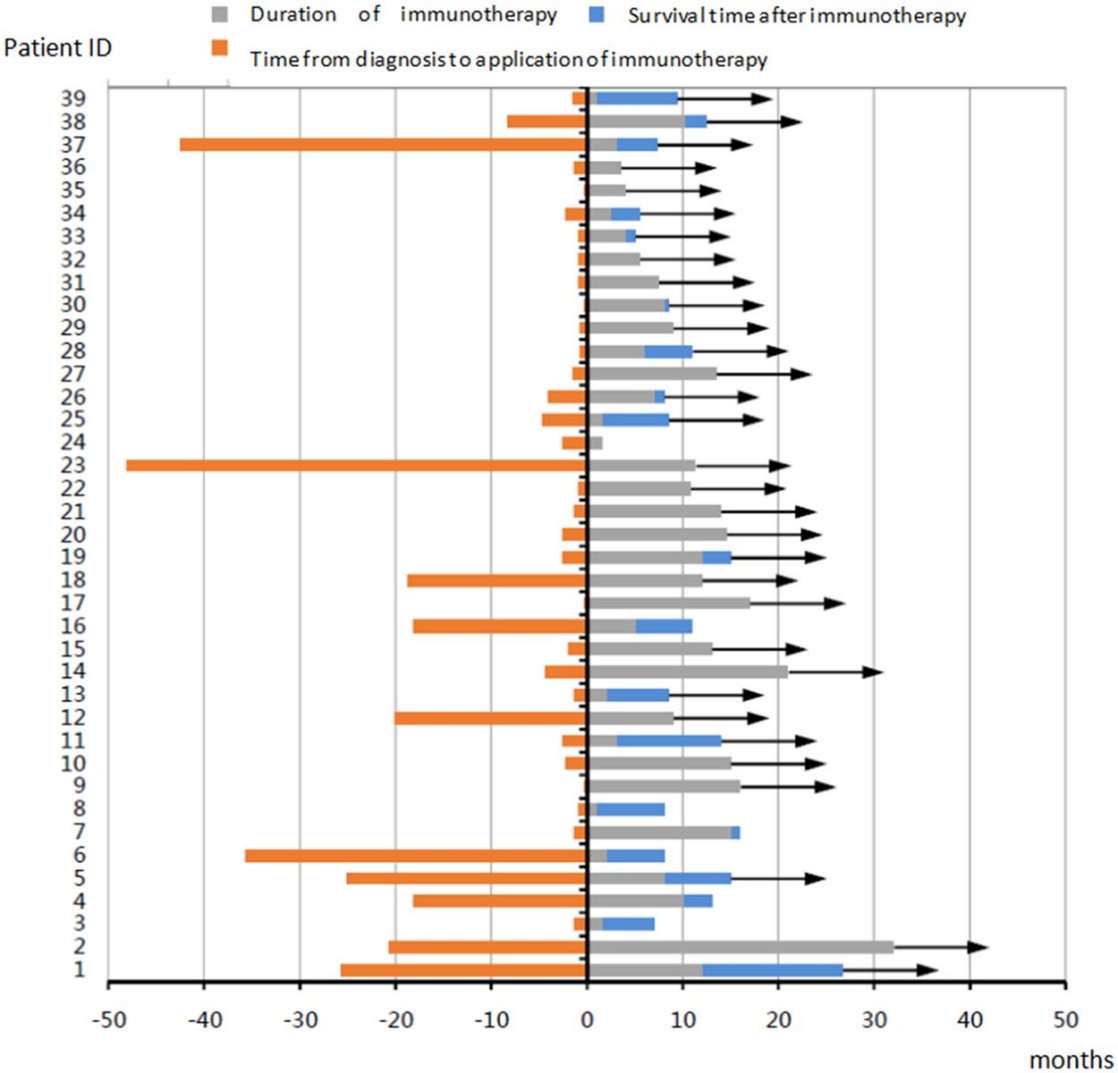
Follow-up of 39 patients (→indicates still living).

The patient’s time from diagnosis to initiation of immunotherapy ranged from 0.5 to 41 months. The median duration of immunotherapy was 8 months (1 to 32 months). One patient with an unknown medication period was removed, and the other patients received 415 cycles of treatment in total; the longest treatment time was 37 cycles (Fig. 1).

Three patients were treated with pembrolizumab and ended their treatment after PR; no other treatment was initiated. Among them, two patients did not progress in the 15 months since treatment finished. One patient progressed 2 years after discontinuation of treatment, after which they received atezolizumab treatment and local radiotherapy for bone metastasis.

### Short-term efficacy analysis

This study included 39 cases, with one case of CR (1.6%), 10 cases of PR (25.6%), 16 cases of SD (41.0%), and 12 cases of PD (30.8%). There was 1 case (2.5%) of pseudo-progression. The ORR was 28.2%, and DCR was 69.2%.

Among the 10 patients over 70-years-old, 4 patients experienced PR and 6 patients experienced SD. Among these patients, the 6 patients who were 75-years-old and older experienced PR in 3 cases and SD in 3 cases. No adverse reactions of grade III or above occurred in these patients.

For brain metastases and the evaluation of the effectiveness of immunotherapy, there were 5 cases of brain/meningeal metastasis. One patient had brain metastasis and meningeal metastasis, three patients had brain metastasis, and one patient had meningeal metastasis. Four patients with brain metastases had neurological symptoms. After immunotherapy, based on intracranial lesion imaging, there were 2 cases PR, 1 case of SD, and 1 case of PD. One patient with meningeal metastasis was relieved of clinical symptoms after immunotherapy. ORR of patients who received immunotherapy for intracranial metastasis was 40%, and DCR was 80%.

Four patients with EGFR mutations were detected, including three patients with therapy-sensitive mutations. There was one case of EGFR exon 19 deletion, two cases of exon 21 L858R mutation, and one case of a non-sensitive mutation of exon 21 residue 861. Three patients with therapy-sensitive mutations were treated with EGFR-TKI drugs as first-line treatment. Two patients underwent secondary biopsy after the development of drug resistance, and the EGFR T790M status was negative. None of these three patients underwent PD-L1 testing. Three patients were treated with immunotherapy as third-line treatment; two patients were treated with pembrolizumab, and one patient was treated with atezolizumab. Finally, two patients showed SD, one patient achieved PR, and ORR for patients with an EGFR mutation was 33.3%.

Statistical analysis showed that age was associated with disease control rate (DCR), while sex, histological type, ECOG score, stage, PD-L1 expression status, how many treatment lines had been completed prior to the initiation of immunotherapy, and smoking were all independent of ORR and DCR. (Table 2).

**Table 2:** Correlation between clinical features and short-term efficacy in 39 patients with non-small cell lung cancer.

	Quantity	CR	PR	SD	PD	ORR	ORR (P value)	DCR	ORR (P value)
**Sex**									
Male	28	1	8	11	8	32.1%	0.461	71.4%	0.709
Female	11	0	2	5	4	18.2%		63.6%	
**Age**									
< 70	29	1	6	10	12	24.1%	0.424	58.6%	0.017
≥70	10	0	4	6	0	40.0%		100%	
**ECOG PS scores**									
0	17	1	3	8	5	23.5%	0.484	70.6%	0.388
1	19	0	7	7	5	36.8%		73.7%	
2	3	0	0	1	2	0%		33.3%	
**Stage**									
III	11	1	4	3	3	45.5%	0.426	72.7%	0.072
IVA	18	0	4	11	3	22.2%		83.3%	
IVB	10	0	2	2	6	20.0%		40.0%	
**Smoking history**									
Never	12	0	2	8	2	16.7%	0.725	83.3%	0.168
Yes	27	1	8	8	10	33.3%		62.9%	
**Pembrolizumab initiation (line of therapy)**									
1	26	1	7	10	8	30.8%	0.625	66.7%	0524
2	7	0	1	3	3	14.3%		60.0%	
≥3	6	0	2	2	2	33%		87.5%	
**PD-L1 status**									
Positive	19	0	5	8	6	26.3%	0.800	68.4%	0.787
Negative/Unknown	20	1	5	8	6	30.0%		70.0%	
**Histological subtype**									
Adenocarcinoma	18	0	5	7	6	26.3%	0.969	68.4%	0.763
Non-Adenocarcinoma	21	2	6	8	5	30.0%		70.0%	

### Long-term survival

**Figure 2:**
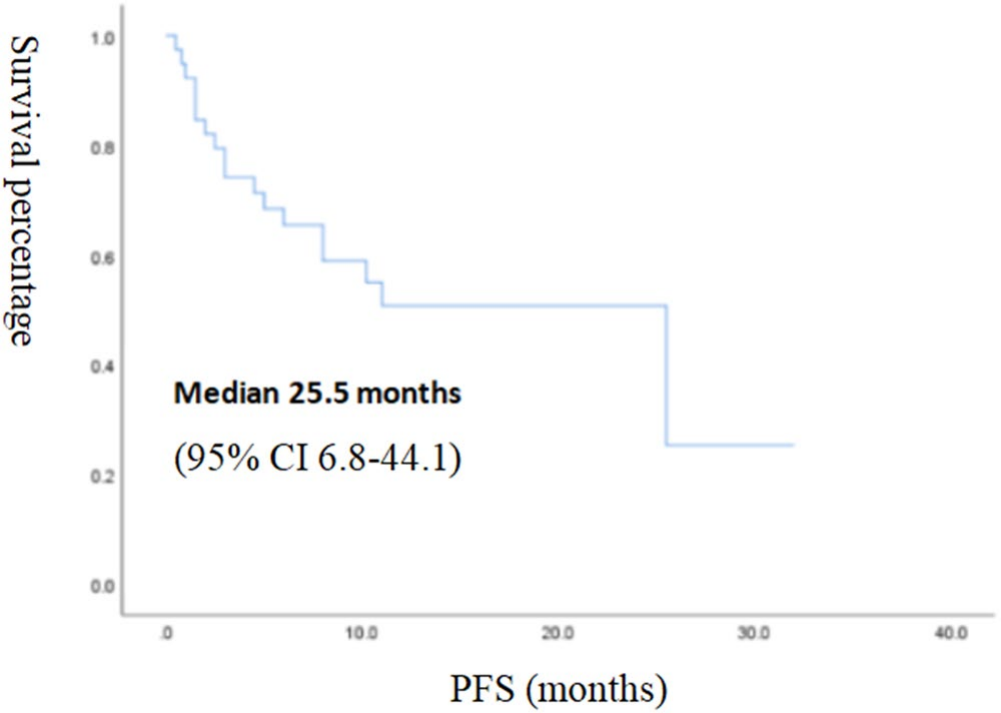
Kaplan-Meier survival curve of PFS in 39 patients.

**Figure 3:**
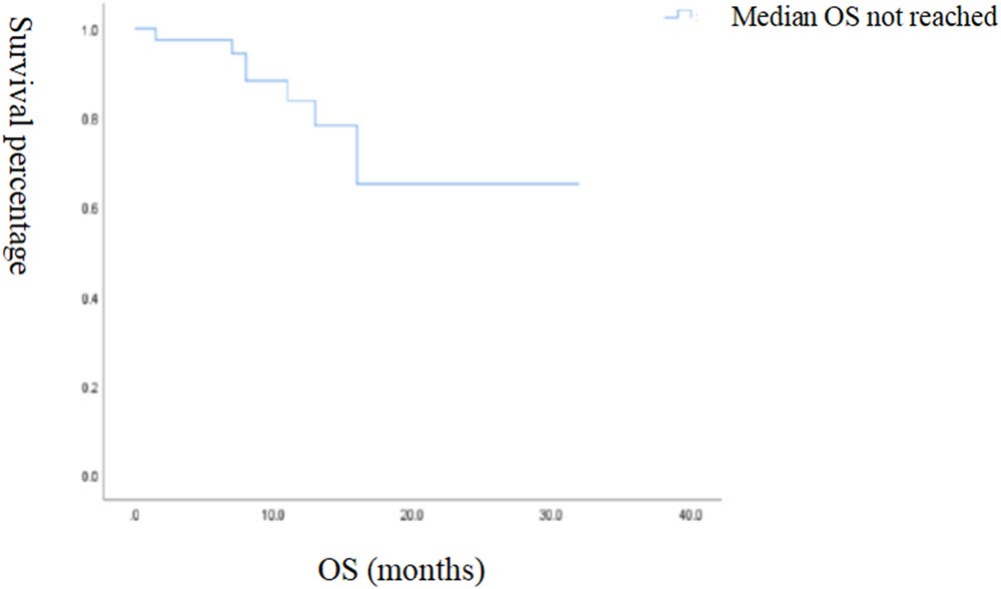
Kaplan-Meier survival curve of OS in 39 patients.

The median follow-up time was 11 months (95% CI 8.2–13.8 months); 7 patients (18.9%) had died at the time of follow-up, 21 patients (53.8%) remained at the progression-free stage, and the longest progression-free time was 32 months. The median PFS was 25.5 months (95% CI 6.8–44.1 months) at follow-up, which was lower than the median OS (Figs 2, 3).

**Figure 4:**
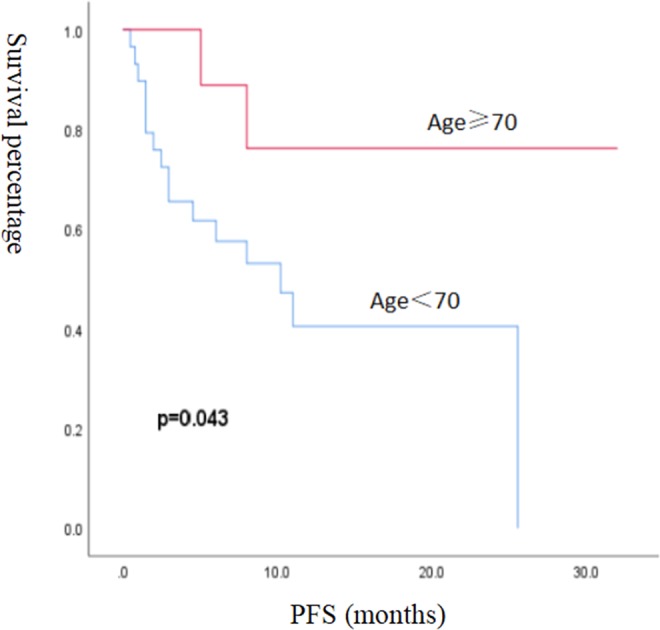
Kaplan-Meier survival curves for PFS were compared among 39 patients with different age groups.

**Figure 5:**
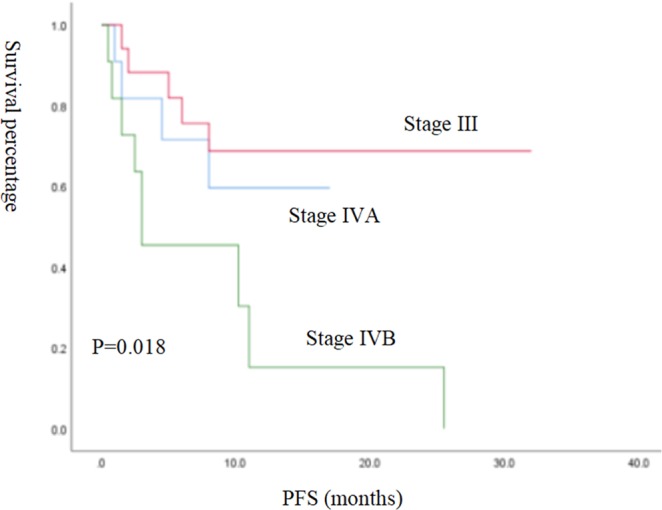
Kaplan-Meier survival curves were used compared to compare PFS in 39 patients with different disease stages.

Because the median OS was not reached in this study, prognostic factors were analysed by PFS. The Kaplan-Meier method was used to analyse the effects of single factors such as age, pathological type, sex, stage, ECOG score, how many treatment lines had been completed prior to the initiation of immunotherapy, smoking history, and PD-L1 detection on PFS. The results showed that age (p = 0.043) and disease stage (p = 0.018) were significantly correlated with PFS (Figs 4, 5). Other factors, such as sex (p = 0.68), pathology (p = 0.581), ECOG score (p = 0.448), how many treatment lines had been completed prior to the initiation of immunotherapy (p = 0.990), smoking history (p = 0.924), and PD-L1 expression status (p = 0.346), have no significant effect on PFS. Important clinical factors with statistically significant effects on prognosis based on univariate analysis were included in the Cox multivariate analysis, and the results showed that age was the only statistically significant factor for PFS (Table 3).

**Table 3:** Factors that affect PFS based on Cox multivariate regression analysis.

FACTOR	PFS		
	HR	95% CI	P value
Age	6.929	1.376–31.938	0.018
Disease stage	0.773	0.225–2.338	0.648
Sex	0.382	0.138–1.313	0.509
Histological subtype	0.426	0.111–2.319	0.137
PD-L1 status	0.581	0.269–10.251	0.581

### Random forest statistical model to predict the prognosis of patients with lung cancer

Considering that the sample size of this study is small, it may not meet the requirements for survival analysis. Therefore, the RF model was used to predict the prognosis of lung cancer patients and the factors affecting prognosis.

The survival of the patients was used as the dependent variable, and variables such as sex, pathological type and age were used as independent variables to construct the prediction model. The out-of-bag estimates calculated the error rate of the model to be 16.22%, and the prediction accuracy rate was 83.78%. The original sample retrograde prediction accuracy rate reached 100%, indicating that the predictive variable of this study has a stable correlation with the survival state of patients after immunological checkpoint inhibitor treatment. The results of the confusion matrix are shown in Tables 4 and 5. The importance scores of the predictors on the survival status are shown in Table 6. As the score increase, the contribution of the independent variable to the prediction of the dependent variable also increases.

**Table 4:** OOB cross-validation model.

		Forecast status	
		Death	Alive
Actual state	Dead	2	5
	Alive	1	29

**Table 5:** Sample back-testing model.

	Forecast status		
	Death	Alive	
Actual state	Dead	7	0
	Alive	0	30

**Table 6:** The importance of the variable predicting the survival status.

Order of importance	Variable	Importance score
1	Disease stage	1.081
2	Smoking history	0.934
3	Pembrolizumab initiation(line of therapy)	0.931
4	Age	0.606
5	Histologicalsubtype	0.604
6	Sex	0.556
7	PD-L1 status	0.554
8	ECOG Score	0.519

## Discussion

This study included 39 patients with advanced NSCLC who underwent PD-1/PD-L1 treatment, with a median follow-up of 11 months, until May 1st, 2018. The data were collected in a real-world situation and show a median PFS of 25.5 months, which was lower than the median OS. The survival time is significantly better than currently published clinical trial data^4–8^. In theory, the population of patients with poor prognosis in real clinical practice is higher, and the prognosis should be relatively worse than clinical trial results. From the real-world survival data released by other countries, the results obtained are similar to or slightly lower than the clinical trial results, which is in line with the prognosis of expected survival^10–13^. However, the survival data obtained in this study are inconsistent with previous studies. We hypothesize the reasons for this result as follows:

1. In this study, outpatients with PD-1/PD-L1 routine treatment experienced relatively high efficacy and were in good general condition, while those with poor efficacy may have been lost to follow-up;
2. Patients with disease progression received other treatments, including radiotherapy and chemotherapy (n = 7), targeted therapy (n = 6), or continued immunotherapy after changing from immunotherapy (n = 3);
3. Patients with male sex (71.7%), a smoking history (69.2%), squamous cell carcinoma (48.7%), immunotherapy as first-line treatment (66.7%), and PD-L1 positive status (48.7%), which are potential survival benefit factors, were present in high proportions in our study^14–16^;
4. Patients who had conditional overseas treatment and long-term use of immunotherapy at their own expense tended to be in a good economic situation, so effective nutritional support and comprehensive nursing could be received, which promotes longer survival time and better prognosis. It has been suggested that the PD-1/PD-L1 inhibitor has a better therapeutic effect in real world populations, and this medicine has promising clinical effects.

Lung cancer incidence increases with age, and the peak of lung cancer diagnosis occurs at 65–74-years-old.With the trend of ageing, it is expected that more elderly patients will be diagnosed in the future^17^, and it is of great importance to explore whether immunotherapy has a definite survival benefit for the elderly. In theory, an increase in age is accompanied by a decline in immune system function, especially T-cell-mediated immune decline, which may lead to poor response to ICI treatment in older populations^18^. The existing results of Phase III clinical trials are inconsistent, and people over the age of 75 are rarely included in clinical trials, so the effectiveness of ICIs in this population is not fully understood. This study included 10 patients over 70-years-old. Statistical analysis showed that patients ≥70-years-old had better DCR and PFS than those under 70-years-old. Among them, 6 patients aged 75 years and older achieved PR in 3 cases and SD in 3 cases. The ORR was 50%. Although there are no randomized controlled clinical trials for older patients, combined with the results of this study and the abovementioned systematic analysis, we can hypothesize that patients over the age of 70 can benefit from ICIs, and people over the age of 75 need to be evaluated as larger populations. In particular, in this study, the over 70-year-old group benefited more than the group of patients who were less than 70-years-old. The over 70-year-old group only represents the dominant population of this study. It is not representative of the population and may be a random phenomenon caused by the small sample, which was enriched with 6 elderly patients with long-term survival benefits.

Brain is the most common metastatic site of NSCLC, and brain metastases occur in approximately 30% of NSCLC patients^19^. Patients with brain metastases have poor prognosis. The median OS of NSCLC with brain metastases with or without treatment is 4–15 months or 4–10 weeks, respectively^20^. The incidence of brain metastasis is expected to increase further as targeted therapy and immunotherapy prolong survival time. How to address brain metastases and to improve the prognosis of brain metastasis through systemic treatment has become a clinical challenge for physicians. Phase III clinical trials of ICIs have excluded NSCLC patients with brain metastases, and there is still insufficient data to support the efficacy of ICIs for brain metastases. This study included five patients with brain/meningeal metastasis in total, and there were two patients with intracranial lesions, two patients with SD, and one patient with PD. ORR was 40%. The survival times of the five patients were 29 months, 15 months, 19 months, 13 months, and 11 months. A previous study in Italy included 1588 non-squamous NSCLC patients^21^, including 409 patients with asymptomatic and symptom-stable secondary brain metastases. The results showed that nivolumab had an ORR of 17%, a DCR of 40%, and the effectiveness was comparable to that of the entire population. The median OS of brain metastasis patients was 8.6 months in this study, while the entire population was 11.3 months. Despite the small sample size, our data and the abovementioned results suggest that brain metastatic NSCLC can benefit from immunotherapy, and our data showed that patients with intracranial lesions have high ORR, which may be related to the history of local radiotherapy in five patients before immunotherapy. Studies have shown that radiotherapy may increase the expression of PD-L1 in tumour lesions. Radiotherapy can also activate dendritic cells and enhance antigen presentation, which in turn helps to improve the killing effect of immune cells; additionally, radiotherapy can help to open the blood-brain barrier and increase the local PD-1 drug concentration in the brain^22^. Based on the above results, immunotherapy with single or combination therapy is effective for intracranial lesions in patients with brain metastases. Immunotherapy is expected to be used as a systemic therapy to control brain metastases after chemotherapy and targeted therapy. Local radiotherapy can improve the efficacy of immunotherapy, and we expect prospective research to collect more data on the combination of local radiotherapy and immunotherapy.

Several clinical trials (CheckMate 057 and KEYNOTE-010) have found that EGFR/ALK-mutant NSCLC is not sensitive to treatment with immunotherapy^4,6^. A common reason is that in populations with EGFR, BRAF, MET, ALK and other gene mutations, it is not easy to produce new antigens of tumours, and the tumour mutation load is lower^23–25^. In this study, four patients with EGFR mutations were included, including one exon 19 deletion of EGFR, two L858R mutations in exon 21, and one non-sensitive mutation of exon 21 residue 861 mutation. Three patients with sensitive mutations were treated with EGFR-TKI drugs in first-line treatment. When drug resistance occurred, two patients were negative for EGFR T790M. Without testing for PD-L1, two patients were treated with pembrolizumab and another was treated with atezolizumab. The results showed that two patients had SD and one had PR. The ORR was 33.3%. In this study, patients with EGFR-sensitive mutations were more sensitive to immunotherapy, suggesting a potential benefit for EGFR mutation-positive NSCLC from ICIs in a clinical setting. Screening for EGFR-positive, TKI-resistant NSCLC patients will further expand the population that may benefit from ICIs.

At present, the exploration of molecular markers among the population receiving ICIs has not been unified, and the detection technology is complex and costly. This study was based on clinical features in order to find more convenient and economical efficacy indicators. However, the results showed no significant influence of sex, pathological type, stage, ECOG score, how many treatment lines had been completed prior to the initiation of immunotherapy, smoking, PD-L1 expression status or other factors on PFS. The possible reasons for this result may be the small sample size of this study, which failed to meet the requirements of survival analysis. Furthermore, it may be due to the limitations of the statistical method itself. The traditional statistical classification method is linear in nature and has many limitations. When the model has a collinear relationship with independent variables, the model parameters are estimated to be unstable. Thus, this study further included the effects of randomized forest prediction variables on survival. The results showed the importance of factors affecting survival were staging, how many treatment lines had been completed prior to the initiation of immunotherapy, smoking history, pathological type, age, PD-L1 expression, sex, and ECOG score, in that order. It provides an intuitive and simple variable model for the selection of populations that may be more suitable for immunotherapy.

Limitations of this study still exist. The follow-up time was short; thus, the OS was not available. Follow-up will be continued in future work. The pathological specimens before treatment were not collected for molecular detection. However, this study innovatively introduced the random forest statistical model to calculate the order of the influence of each predictor on survival and presented the order of importance of factors related to prognosis, which has important clinical application value.

## Data Availability

The datasets generated and analyzed during the current study are available from the corresponding authors on reasonable request.
