# Author Correction: Real-world evidence and clinical observations of the treatment of advanced non-small cell lung cancer with PD-1/PD-L1 inhibitors

**DOI:** 10.1038/s41598-020-58487-5

**Published:** 2020-01-27

**Authors:** Peng Song, Jingcheng Zhang, Congcong Shang, Li Zhang

**Affiliations:** 1 Department of Respiratory Medicine, Peking Union Medical College Hospital, Chinese Academy of Medical Sciences & Peking Union Medical College, Beijing, China; 2 Department of Internal Medicine, Peking Union Medical College Hospital, Chinese Academy of Medical Sciences & Peking Union Medical College, Beijing, China; 3 Department of Allergy, Henan Provincial People’s Hospital, Zhengzhou, China

Correction to: *Scientific Reports* 10.1038/s41598-019-40748-7, published online 12 March 2019

The original version of this Article contained an error in the title of the paper.

“Real-world evidenceand clinical observations of the treatment of advanced non-small cell lung cancer with PD-1/PD-L1 inhibitors”

now reads:

“Real-world evidence and clinical observations of the treatment of advanced non-small cell lung cancer with PD-1/PD-L1 inhibitors”

This has now been corrected in the PDF and HTML versions of the Article.

